# Healthcare leaders navigating complexity: a scoping review of key trends in future roles and competencies

**DOI:** 10.1186/s12909-024-05689-4

**Published:** 2024-07-03

**Authors:** Samantha Spanos, Elle Leask, Romika Patel, Michael Datyner, Erwin Loh, Jeffrey Braithwaite

**Affiliations:** 1 Centre for Healthcare Resilience and Implementation Science, Australian Institute of Health Innovation, Macquarie University, 75 Talavera Rd, North Ryde, Sydney, NSW 2109 Australia; 2 Royal Australasian College of Medical Administrators, Melbourne, VIC Australia

**Keywords:** Leadership, Physician leadership, Future health care, Future healthcare, Training, Complex systems

## Abstract

**Background:**

As healthcare systems rapidly become more complex, healthcare leaders are navigating expanding role scopes and increasingly varied tasks to ensure the provision of high-quality patient care. Despite a range of leadership theories, models, and training curricula to guide leadership development, the roles and competencies required by leaders in the context of emerging healthcare challenges (e.g., disruptive technologies, ageing populations, and burnt-out workforces) have not been sufficiently well conceptualized. This scoping review aimed to examine these roles and competencies through a deep dive into the contemporary academic and targeted gray literature on future trends in healthcare leadership roles and competencies.

**Methods:**

Three electronic databases (Business Source Premier, Medline, and Embase) were searched from January 2018 to February 2023 for peer-reviewed literature on key future trends in leadership roles and competencies. Websites of reputable healthcare- and leadership-focused organizations were also searched. Data were analyzed using descriptive statistics and thematic analysis to explore both the range and depth of literature and the key concepts underlying leadership roles and competencies.

**Results:**

From an initial 348 articles identified in the literature and screened for relevance, 39 articles were included in data synthesis. Future leadership roles and competencies were related to four key themes: innovation and adaptation (e.g., flexibility and vision setting), collaboration and communication (e.g., relationship and trust building), self-development and self-awareness (e.g., experiential learning and self-examination), and consumer and community focus (e.g., public health messaging). In each of these areas, a broad range of strategies and approaches contributed to effective leadership under conditions of growing complexity, and a diverse array of contexts and situations for which these roles and competencies are applicable.

**Conclusions:**

This research highlights the inherent interdependence of leadership requirements and health system complexity. Rather than as sets of roles and competencies, effective healthcare leadership might be better conceptualized as a set of broad goals to pursue that include fostering collaboration amongst stakeholders, building cultures of capacity, and continuously innovating for improved quality of care.

**Supplementary Information:**

The online version contains supplementary material available at 10.1186/s12909-024-05689-4.

## Background

Healthcare leadership has grown in scope and importance in response to the increasing complexity of healthcare delivery [1]. Healthcare systems have become increasingly multifaceted, delivering a vast array of services across multiple levels, from preventative and primary care to acute, specialized care, and long-term care, to address the care needs of a changing population [1]. As populations age, chronic diseases rise, and the epidemiology and demographics of disease shift, new models of care rapidly emerge to address the ever-expanding spectrum of patient needs [2]. Advancements in technologies, tests and treatments and personalized medicine come with regulatory and ethical implications, and a growth in workforce specializations [3, 4]. Healthcare leaders are navigating evermore complex webs of actors in the system – doctors, nurses, technicians, administrators, insurers, and patients – striving to balance priorities, foster collaboration, and provide strategic direction toward high-quality and safe patient care [5]. At the same time as running complex services, healthcare leaders need to continually assess, implement, and govern new technologies and services, adhere to the latest regulations and guidelines, operate within the confines of budgetary allocations, and meet growing consumer expectations for affordable and accessible care [6, 7]. 

Competent healthcare leadership is widely considered to be critical for improving patient safety, system performance, and the effectiveness of healthcare teams [8–10]. Leadership has been identified as a key shaping influence on organizational culture [11], including workplace commitment to safety [12], and on preventing workforce burnout [13, 14]. The increased need for multidisciplinary and integrated care models has shed growing light on the leadership roles of clinicians, including physicians, nurses, and allied health practitioners [15–17]. Individuals with both clinical and leadership expertise have been considered vital in complex healthcare landscapes because of their ability to balance administrative needs while prioritizing safety and high-quality care provision [18–22]. For example, physician leaders, through their deep understanding of clinical care and their credibility and influence, have been considered best able to devise strategies that improve patient care amidst stringent financial conditions [23–26]. Clinical leaders, particularly physician leaders, might also be of key importance for facilitating the success of collaborative care and care integration [27]. 

The formalization of healthcare leadership emerged as the importance of specialized healthcare leadership skills became increasingly needed, recognized and understood [1, 28, 29]. Leadership in healthcare has been conceptualized in several different ways, and a multitude of theories, frameworks, and models have been proposed to explain leadership roles and responsibilities [30–33]. For example, the CanMEDS Framework describes the Leader Role of physicians, which is comprised of key and enabling competencies, tasks, and abilities [34, 35], and adaptations to this Framework emphasize the varying roles that leadership comprises and the competencies that fulfill them [36]. Although these frameworks present a good starting point for articulating leadership role scopes and their associated competencies, many fall short in explaining how leaders navigate complex, dynamic, multi-dimensional, and highly variable healthcare systems [37]. This is becoming increasingly recognized; CanMEDS is due to be updated in 2025 to incorporate competencies related to complexity [38]. Meanwhile, on the front lines, lack of role clarity and ambiguity about tasks and responsibilities presents a significant barrier for healthcare leaders [1, 15]. In complex and unpredictable systems like healthcare, leaders spend substantial time ‘sense-making’, understanding, prioritizing and responding adaptively according to the needs of the situation [39, 40]. The latest research on future healthcare trends tells us that increasing complexity associated with digital innovation, healthcare costs, regulatory compliance, sustainability concerns and equitable resource distribution will pose challenges to all actors in health systems [41–45]. In the face of these emerging challenges, it is vital to understand the range and type of roles and competencies that leaders will need to fulfil in the imminent future.

The aim of this scoping review is to examine the literature on the key trends in roles and competencies required for healthcare leaders in the future. We conceptualized ‘competencies’ as the attributes, skills, and abilities that comprise the fulfilment of varying leadership roles, as informed by the CanMEDS Framework [34, 36]. Scoping review methodology was utilized to capture a broad range of literature types and identify key themes or groupings of future trends in leadership roles and competencies. Rather than focusing on answering specific questions (as per previous systematic reviews on leadership [46, 47]) or developing theory (by utilizing a theoretical review approach to leadership literature [48, 49]), we sought to map and identify patterns and trends within the leadership literature [50]. To investigate trends in leadership roles and competencies, we targeted emerging perspectives from key reputable thought leaders to supplement academic research [51, 52]. 

## Methods

The conduct and reporting of this review followed the Preferred Reporting Items for Systematic Reviews and Meta-Analyses Extension for Scoping Reviews (PRISMA-ScR) guidelines [53]. 

### Search strategy

Comprehensive search strategies were developed, adapting search strategies utilized in a previous systematic review on physician leadership [26], and receiving input and expertise from two clinical librarians at Macquarie University (see supplementary file 1 for database search strategies). Medline, Embase, and Business Source Premier were searched from January 2018 to February 2023 to enable meaningful inferences to be made about *future trends* based on current perspectives. To capture key trends, patterns, shifts, and forecast changes to healthcare leadership, the Medline database search was limited to the ‘Trends’ subheading, “*used for the manner in which a subject changes, qualitatively or quantitatively, with time, whether past, present, or future. Includes “forecasting” & “futurology"*” (see supplementary file 1) [54]. For Embase and Business Source Premier, the ‘Trends’ subheading was not available, and instead key search terms were included to capture future trends, including “predict*”, “forecast*”, “shift*” and “transform*”. Efforts were made to locate texts that could not be retrieved, by searching Macquarie University’s digital library records and contacting authors to request the full text.

To complement the database searches, targeted searches of the Faculty of Medical Leadership and Management (FMLM; UK) website and The King’s Fund (UK) website were undertaken to identify emerging perspectives on the future roles and requirements of healthcare leaders. Targeted website searches can aid in uncovering unpublished yet relevant research identified by advocacy organizations or subject specialists, and research potentially missed by database searches [52, 55]. Key search terms entered into the websites included ‘future healthcare’, ‘medical leader’, ‘clinical leader’, ‘medical manager’, ‘physician executive’, and ‘education and training’. We included articles that focused on leaders with a clinical background and leaders without a clinical background, to provide a comprehensive overview of leadership roles and requirements of reference to health systems [26]. 

### Article selection

#### Database literature search

References were uploaded into online data management software Rayyan [56], and duplicate records were identified and removed. Titles and abstracts of results were screened by three team members (SS, EL, RP) according to the inclusion and exclusion criteria (Table 1). Articles were included if they focused on future trends in the roles, competencies, attributes, or requirements of healthcare leaders, and if they reported on countries within the Organization for Economic Co-operation and Development (OECD). We limited our search to OECD countries to maximize the generalizability of findings within a developed context and enable meaningful trends to be identified. A subset of the articles was screened by all three team members to ensure that decisions were being made in a standardized manner. After this article subset was screened, the three team members discussed screening decisions, and disagreements were resolved by consensus or through discussion with JB [57]. During this process, two further exclusion criteria (#4 and #5, Table 1) were added to ensure that the screening process adhered to the aim of the current review. We excluded articles that focused on theories and definitions of leadership (e.g., for the purpose of developing educational or professional frameworks) without highlighting trends or changes in roles and competencies for future leadership. We also excluded articles that focused on healthcare interventions in which leaders may have been participants, but their roles or competencies were not the focus. Articles included at title and abstract screening were independently read in full and assessed for eligibility. Disagreements about inclusion were resolved through discussion, with JB available for arbitration if necessary. It was determined at this stage that if articles were conference abstracts in which the full presentation could not be accessed, the article of focus was sought and included in the analysis.

**Table 1:** Inclusion and exclusion criteria for literature screening

**Inclusion criteria:**
1. Articles that report on comparable international health systems or contexts (i.e. OECD countries);
2. Articles that focus on senior leaders, managers, or administrators in healthcare;
3. Articles that focus on roles, competencies, attributes, or requirements of healthcare leaders;
4. Articles that focus on future healthcare leadership roles or requirements, including those that report on forecast changes, trends, and shifts in roles, competencies, skills, or requirements.
**Exclusion criteria:**
1. No full-text available;
2. Not in English language;
3. Published prior to 2018;
4. Articles that focus on distinguishing definitions or theories of healthcare leadership without focusing on forecast changes, trends and shifts in roles, competencies, skills, or requirements for future healthcare leadership;
5. Articles that discuss healthcare leadership in the context of a specific intervention, program, or initiative without focusing on future roles or requirements of healthcare leaders.

OECD, Organization for Economic Co-operation and Development

#### Targeted gray literature search

References were screened according to the inclusion and exclusion criteria (Table 1), except that articles only needed to report (rather than focus) on future leadership roles and requirements. This is because we wanted to ensure that our analysis broadly captured the most recent sources of information on healthcare leadership requirements, even if these sources did not focus exclusively on leadership.

### Data charting process

Data from all records were appraised and charted simultaneously using a purpose-designed Excel data charting form designed by SS (and subsequently reviewed and endorsed by RP and EL). Multimedia records arising from targeted gray literature searches were listened to and transcribed by RP and checked by SS. Extracted data included article details (authors, year, country, text type), leadership focus (training or educational approaches, styles of leadership), and major and minor themes. Database literature were extracted first to identify and develop themes, and the targeted gray literature were extracted second to extend and embellish those themes.

### Synthesis of results

Data from included articles were synthesized according to the Arksey and O’Malley framework for scoping reviews, selected for its detailed guidance on data collation, synthesis, and presentation [58]. The breadth, range, and type of data were analyzed using descriptive statistics, and underlying groups of leadership roles and competencies were analyzed using thematic analysis. First, the authorship team familiarized themselves with the articles to gain a broad overview of contexts in which leadership was discussed. An inductive approach was used to identify emerging themes of leadership roles and competencies in the database literature, where common concepts were identified, coded, and grouped together to form themes. Team discussion facilitated the final set of themes that were interpreted from the data. During this process, the extracted data were compared to the codes, groups, and resultant themes to examine the degree of consistency between the data and the interpreted findings. Where inconsistencies were identified, suggested changes (e.g., to code labels or groupings) were compared, and the most appropriate changes adopted. Targeted gray literature sources were deductively analyzed according to the identified themes.

## Results

### Selection of sources of evidence

**Fig. 1:**
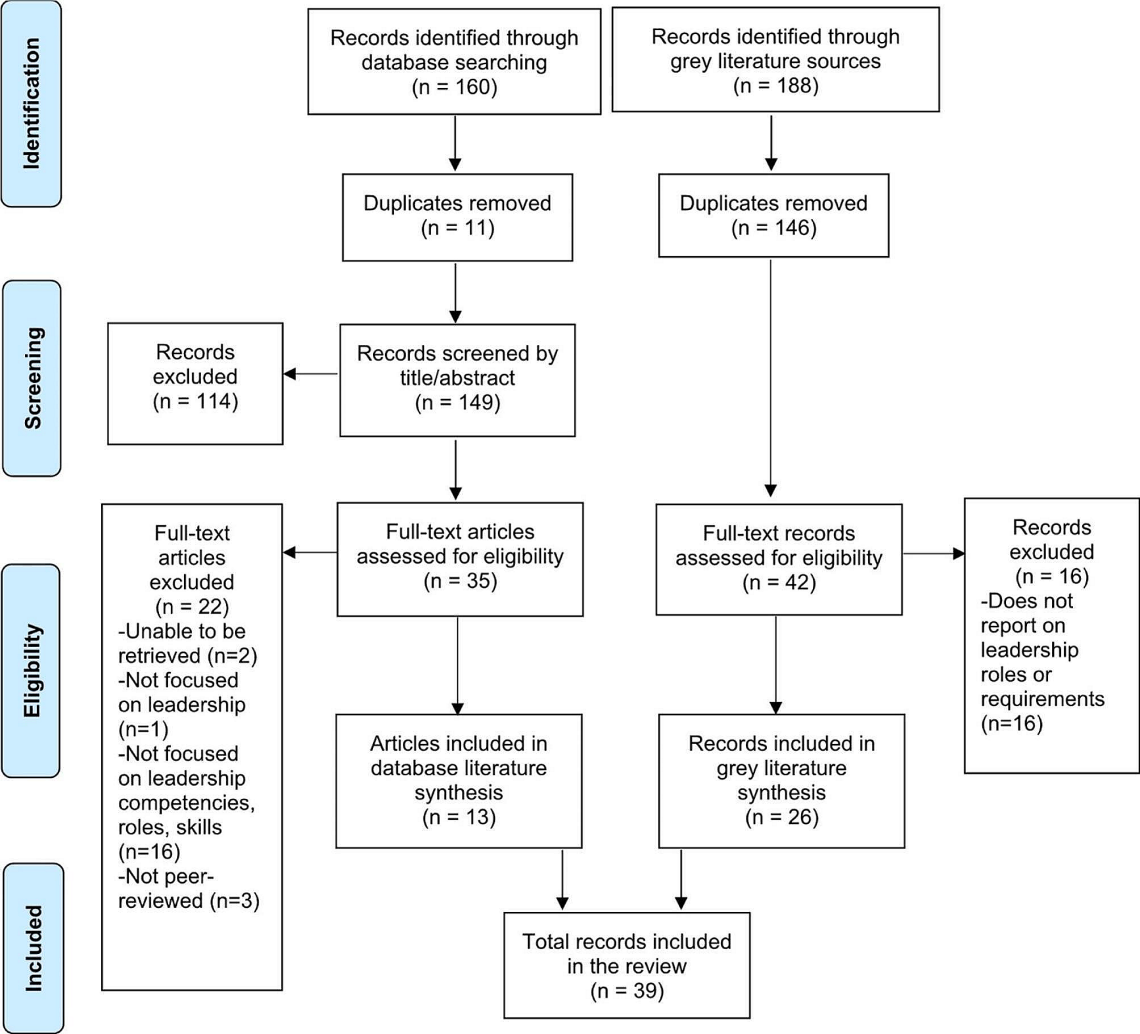
PRISMA flowchart displaying the process of identification and selection of included articles

Figure 1 displays the process of identification and screening of included studies. Database searches yielded 160 records, from which 11 duplicates were removed. The remaining 149 database records were screened by title and abstract, after which a further 114 records were excluded. Of the remaining 35 that were assessed for eligibility, 22 were excluded, and 13 were included in the current review. Targeted gray literature searches yielded an additional 188 records, from which 146 were identified as duplicates and removed. The remaining 42 records were read in full and assessed for eligibility, from which a further 16 were excluded, and 26 were included in the current review. In total, 39 records were retained and synthesized.

### Characteristics of sources of evidence

The characteristics of the included records are displayed in Tables 2 and 3. Of the database literature, most articles were published in the USA (*n* = 11), and the remaining two articles were published in Canada and Australia. Seven articles were empirical; three studies employed qualitative methods [59–61], three were quantitative [62–64], and one mixed methods [65]. Six articles were non-empirical; three were perspective pieces [66–68], and three were reports on training or organizational interventions [69–71]. Of the targeted literature, blog-type articles were most common (*n* = 11) [72–82], followed by news articles (*n* = 5) [83–87], reports (*n* = 4) [88–91], editorials (*n* = 2) [92, 93], podcasts (*n* = 2) [94, 95], and video and interview transcripts (*n* = 2) [96, 97]. As targeted gray sources selected were The King’s Fund website and the FMLM website, the records from these websites were published in the UK.

**Table 2:** Characteristics of database literature included in scoping review

Author	Country	Article type	Participants and setting	Objective or aim	Themes discussed
Angood (2022) [66]	USA	Non-empirical (perspective)	No participants; non-specific setting	To highlight the importance of personal transitions and transformations for physician leaders.	• Innovation and adaptability • Collaboration and communication • Self-development and self-awareness
Appelbaum (2022) [62]	USA	Empirical (quantitative)	Dual medical and business; primary care, hospital, academia, start-up, consulting, pharmacy, other	To determine the business competencies and training applicable throughout different stages of physician leaders’ careers.	• Innovation and adaptability • Collaboration and communication
Best (2020) [37]	Australia	Empirical (qualitative)	Medical specialists; hospital	To identify and explore the implications of different leadership approaches for future implementation of clinical genomics.	• Innovation and adaptability • Collaboration and communication
Borgwardt (2019) [71]	USA	Non-empirical (report)	No participants; large integrated care organization	To describe the process, components, and success of a leadership-driven change management initiative.	• Innovation and adaptability • Collaboration and communication
Brace (2020) [67]	USA	Non-empirical (perspective)	No participants; non-specific setting	To explore emerging opportunities for healthcare leaders, how they can best adapt to changing roles, and how they can remain relevant and effective.	• Innovation and adaptability • Collaboration and communication • Self-development and self-awareness
Crabtree (2020) [60]	USA	Empirical (interviews), non-empirical (review)	Medical, other clinical, and non-clinical staff and leaders; primary care	To explore the leadership attributes that support innovative change in primary care practices.	• Innovation and adaptability • Collaboration and communication • Self-development and self-awareness
Hopkins (2018) [63]	USA	Empirical (quantitative)	Medical specialists; university and hospital	To determine the effectiveness of physician education on leadership competencies when applied to leadership training.	• Innovation and adaptability • Collaboration and communication • Self-development and self-awareness
Ramirez (2021) [69]	USA	Non-empirical (report)	No participants; non-specific setting	To describe the competencies of healthcare managers and the training methods and venues to promote their professional development.	• Innovation and adaptability • Collaboration and communication • Self-development and self-awareness
Sandberg (2022) [64]	USA	Empirical (quantitative), non-empirical (review)	Senior healthcare leaders; hospital, primary care, specialist, nursing home, other	To explore the transitions made by healthcare leaders to continue effective operations during the COVID-19 pandemic.	• Innovation and adaptability • Collaboration and communication • Self-development and self-awareness
Satiani (2022) [65]	USA	Empirical (quantitative)	Medical specialists; primary care, specialist, hospital, emergency, laboratory, pharmacy, rehabilitation	To describe healthcare leaders’ engagement and experience within a faculty leadership program and draw implications for future programs.	• Innovation and adaptability • Collaboration and communication • Self-development and self-awareness
Wain (2020) [70]	USA	Non-empirical (report)	No participants; hospital	To describe the importance of continuing leadership education for healthcare leaders and how learnings can be applied across leaders’ career stages.	• Innovation and adaptability • Collaboration and communication • Self-development and self-awareness
Woiceshyn (2022) [61]	Canada	Empirical (qualitative)	Medical and non-clinical leaders, administrative staff; hospital	To explore the adaptive capacity of physician leaders during the COVID-19 pandemic and the creation of a peer-professional leadership model.	• Innovation and adaptability • Collaboration and communication
Wright (2018) [68]	USA	Non-empirical (perspective)	No participants; non-specific setting	To propose a sequence of actions for physician leaders to effectively develop new skills and behaviors in the context of changing healthcare.	• Innovation and adaptability • Collaboration and communication • Self-development and self-awareness

Abbreviations: USA: United States of America

**Table 3:** Characteristics of targeted gray literature included in scoping review

Author	Country	Article type	Objective or aim	Themes discussed
*The King’s Fund*				
Anadaciva (2020) [72]	UK	Blog	To describe approaches to integrated health care and services and highlight questions for healthcare leaders involved in strategic decisions.	• Collaboration and communication
Bailey (2020) [73]	UK	Blog	To explain the psychological needs of healthcare employees in their workplaces and how leaders can best facilitate these needs.	• Innovation and adaptability • Collaboration and communication
Bailey (2021) [74]	UK	Blog	To identify how healthcare leaders can support solution-driven behaviors to excessive workload for healthcare employees.	• Collaboration and communication • Consumer engagement and advocacy
Collins (2021) [77]	UK	Blog	To describe clinical leadership behaviors that contributed to the rapid planning and re-design of models of care during COVID-19.	• Innovation and adaptability • Collaboration and communication • Consumer engagement and advocacy
Dixon (2018) [79]	UK	Blog	To describe public engagement strategies that can be utilized by healthcare organizations and their leaders to improve future healthcare.	• Collaboration and communication • Consumer engagement and advocacy
Dougall (2018) [88]	UK	Publication	To describe the strategies for creating transformational change within healthcare with a focus on collaborative leadership styles.	• Innovation and adaptability • Collaboration and communication • Consumer engagement and advocacy
Fogden (2022) [89]	UK	Publication	To describe how data can be leveraged to better understand economic and social population needs, with implications for leadership action.	• Innovation and adaptability
Ham (2018) [90]	UK	Publication	To review health system improvement priorities in the context of health service funding and identify the role of healthcare leaders in actualizing change.	• Innovation and adaptability • Collaboration and communication • Consumer engagement and advocacy
Hancock (2018) [96]	UK	Transcript	To highlight the opportunities for healthcare improvement and the role of system leadership in cultivating improvement.	• Innovation and adaptability
Leveson (2021) [80]	UK	Blog	To describe the development of a trainee leadership board providing leadership training, skill development, and experiential learning.	• Innovation and adaptability • Collaboration and communication • Self-development and self-awareness
Naylor (2020) [81]	UK	Blog	To explore the requirements of improving health system sustainability including the role of healthcare leaders.	• Innovation and adaptability • Collaboration and communication
Warren (2022) [95]	UK	Multimedia	To examine the role of healthcare leaders in producing compassionate cultures and promoting staff mental health and well-being.	• Innovation and adaptability • Collaboration and communication
*Faculty of Medical Leadership and Management (FMLM)*				
Baird (2020) [97]	UK	Article	To outline the qualities leaders needs to demonstrate, how they deal with challenges, and the supports they need during and after medical training.	• Innovation and adaptability • Collaboration and communication • Self-development and self-awareness
Bhopal (2018) [75]	UK	Article	To highlight the importance of healthcare leaders familiarizing themselves with fundamental public health principles.	• Innovation and adaptability • Collaboration and communication • Consumer engagement and advocacy
Cheetham (2021) [76]	UK	Article	To examine the importance of junior doctor leadership in the healthcare system during a pandemic, but also thereafter.	• Innovation and adaptability • Self-development and self-awareness
Cottey (2018) [78]	UK	Article	To outline the steps required for leaders to promote teamwork in a healthcare team.	• Collaboration and communication • Self-development and self-awareness
Evans (2020) [92]	UK	Editorial	To promote the *FMLM Navigator* mobile app, a tool supporting leadership development for medical leaders at various career stages.	• Self-development and self-awareness
FMLM (2019) [83]	UK	News	To explain why leadership is an essential skill for doctors of the future, and how doctors will be expected to act as leaders.	• Innovation and adaptability • Collaboration and communication • Consumer engagement and advocacy
FMLM (2021) [84]	UK	Article	To demonstrate the value of doctors completing a fellowship and maintaining engagement with alumni to continue their leadership development journeys.	• Innovation and adaptability
FMLM (2021) [85]	UK	Editorial	To describe the leadership required to support workforce wellbeing post-pandemic.	• Innovation and adaptability • Collaboration and communication
FMLM (2022) [86]	UK	News	To reflect on key themes and challenges of future healthcare and leadership from various international perspectives.	• Innovation and adaptability • Collaboration and communication • Consumer engagement and advocacy
Lakhani (2020) [87]	UK	News	To highlight the role of leaders in supporting a diverse workforce and standing for fairness and justice.	• Collaboration and communication
Lakhani (2021) [93]	UK	Editorial	To examine the importance of diversity in medical leadership.	• Innovation and adaptability • Consumer engagement and advocacy
Moralee (2018) [94]	UK	Multimedia	To highlight the need for medical leaders to develop skills to meet the ever-changing needs of patients and healthcare organizations.	• Innovation and adaptability • Collaboration and communication • Self-development and self-awareness
Swanwick (2020) [91]	UK	Resource	To explain the importance of making leadership learning and assessment explicit in the medical undergraduate curriculum.	• Self-development and self-awareness
Tweedie (2018) [82]	UK	Blog	To describe the attributes of outstanding leaders and how these attributes can be applied.	• Innovation and adaptability

Abbreviations: FMLM: Faculty of Medical Leadership and Management; UK: United Kingdom

### Leadership roles and competencies

All 13 articles derived from the database searches focused on **innovation and adaptation** in future leadership. Two empirical articles reported on the ways in which clinical and non-clinical leaders innovated during the COVID-19 pandemic, rapidly designing new models of hospital care [61] and extending their roles to encompass the implementation of virtual leadership [64]. Qualitative investigations explored the importance of entrepreneurial leadership for implementing clinical genomics [59] and key leadership attributes for practice-level innovation and sustainability [60]. Four articles examined leadership training approaches that build physicians’ capacity to understand, adapt to, and manage change, overcome resistance, and think entrepreneurially [62, 63, 65, 70]. Two reports described the necessity for healthcare leaders to be able to create a shared vision for an organization; one highlighted the importance of leaders being confident and “self-propelled to intervene” [69], and one emphasized physician leaders’ credibility as a catalyst for change management among healthcare providers [71]. The latter report also identified that visible and committed leadership that is sensitive to workplace cultures is critical for the success of change management activities [71]. Three perspective pieces discussed increasing opportunities for medical and other clinical leaders to create positive change in increasingly complex healthcare landscapes and fulfill the demands of the industry and public [66–68]. 

In the targeted gray literature, 19 of 26 records (73%) focused on innovative and adaptive leadership. Records primarily explored adaptive leadership behaviors during COVID-19, such as service redesign, introducing improved flexibility, learning mechanisms, and support platforms [73, 76, 77, 97], and future innovation to manage climate change impacts [81], growing inequities [89], and emerging technologies [75, 83, 94, 96]. Comfort with change, vision setting, and a desire to innovate were emphasized as key leadership attributes for future healthcare [82, 83, 88, 96]. Records also explored how to best train and develop leaders for transforming health systems, including the National Health Service (NHS) [84, 90, 96]. New leadership training structures were proposed that foster innovation and adaptability in leaders [80, 90, 96] and encourage flexibility for cross-disciplinary learning.

**Collaboration and communication** was a second theme that emerged across all 13 database articles. Three studies explored how collaborative leadership can foster innovation with regards to implementing genomics testing [59], creating new work models during COVID-19 [61] and developing new leadership styles via telecommunications [64]. Six articles focused on the importance of collaborating to build relationships across organisations [67, 68, 71] and within teams [65, 69, 70]. Three articles highlighted that effective communication contributes to organizational success, through fostering psychologically safe cultures [60, 66] and generating the trust and rapport necessary for implementing technological innovations [71]. Two studies examined the impact of leadership training on physicians’ communication competencies [62, 63].

In the targeted gray literature, 17 of 26 records (65%) focused on collaboration and communication. Records discussed specific initiatives to improve communication in clinical teams, such as staff surveys, daily huddles, and dedicated days for networking [75, 77, 80, 95]. Cross-boundary collaboration and collective leadership (e.g., between clinicians and managers) [83] were advocated as a means to solve challenges [81, 90], help build public trust [79, 83], and improve quality of care [78, 83, 85, 94]. Twelve records focused on the importance of team and leadership collaboration to create positive workplace cultures and improve staff wellbeing, through communication strategies such as openness and honesty [78, 80, 95], active listening and empathy [73, 78, 86, 88, 90], transparency [88, 94, 95], and inclusivity [85, 94]. Three articles emphasized that encouraging staff autonomy, building trust, and demonstrating compassion facilitate better quality care than demanding and punitive leadership actions [73, 74, 88]. 

Nine of 13 database articles (69%) focused on a third theme, **self-development and self-awareness** in leadership. Four articles examined approaches to leadership development that incorporated self-development and self-awareness (e.g., personality testing) [63, 65, 69, 70], with two articles describing these competencies as enablers for the development of other more advanced competencies (e.g., execution) [69, 70]. Similar competencies explored included landscape awareness [60], self-organisation [60], emotional intelligence [64], and self-examination, the last of which was described as essential to gain skills beyond clinical roles [68], facilitate positive perceptions of others [66], and to remain relevant and effective in a changing healthcare environment [67]. One article also proposed strategies such as journaling, mindfulness, and feedback to encourage ongoing reflection on leadership decisions and biases [67]. 

In the targeted gray literature, seven of 26 records (27%) focused on self-development and self-awareness. Records examinedd the importance of continual personal leadership development, including mentoring and experiential learning, to facilitate understanding of one’s own skills [78, 80, 97]. Tools to facilitate self-reflection in physician leaders were advocated including the FMLM smartphone app [92] and leadership longitudinal assessments [91]. Self-care and resilience practices (e.g., meditation, social support) were also advocated for physician leaders as a means to manage “greater levels of stress and responsibility” [94]. 

**Consumer engagement and advocacy** was a fourth theme and a focus of nine targeted gray literature records (35%). Records discussed patient and community engagement as essential for health system improvement, and examples included involving patients in health service design [74, 77], creating channels of ongoing dialogue [79, 83] and building stronger health system-community relationships [79, 88]. Two records described the importance of public health messaging in improving health literacy [83] and countering misinformation [86], and two focused on the role of leaders in advocating for social justice and striving to improve equitable outcomes [75, 93].

## Discussion

This scoping review identified 39 key resources that explored future trends in healthcare leadership roles and competencies. These records were derived from a combination of academic and targeted gray literature searches, juxtaposed and synthesized to build understanding of leadership to improve health systems into the future. Four themes of competencies emerged from the findings – innovation and adaptation, communication and collaboration, self-development and self-awareness, and consumer engagement and advocacy.

### Leadership roles and competencies

The competencies of healthcare leaders given the most attention in the literature over the last five years relate to **innovation and adaptability**. Both the academic and targeted gray literature focused on how leaders, clinical and non-clinical, demonstrated innovativeness and adapted to the demands of COVID-19, including rethinking and redesigning systems to support staff and patients [64, 77]. The second focus of the literature on innovation and adaptability was geared toward the *development* of these capacities in leaders through education and training, as well as through opportunities for leaders to actualize their skills [70, 90]. The literature indicated that as the complexity of healthcare is accelerating, leaders must both understand, and have opportunities to demonstrate, innovation amidst dynamic, variable, and demanding environments [59, 60, 71]. This aligns with prior research demonstrating that innovation uptake requires strong change management, and the ability to rapidly assess, understand, and apply innovative changes (e.g., medical technologies) [1, 98]. While innovations might improve the system’s ability to deal with complex challenges in the long-term, their implementation can be challenged by a number of moving parts – including workforce changes, new rules and regulations, fluctuating resources and new patient groups – which leaders must consider and appropriately plan for [99, 100]. Perhaps an even greater challenge for leaders to overcome when embracing innovation is the tendency for growing complexity to lock the organization into suboptimal conditions (i.e., inertia) [101]. Building awareness of the interacting components of complex systems and the flexibility required for adaptation and resilience should be a key focus of healthcare leadership education and training [102]. 

Competencies associated with **communication and collaboration** have also been a focus of the healthcare leadership literature. Academic literature dealt primarily with how collaborative structures and behaviors can help leaders innovate and build organizational cultures geared for success [59, 61, 71]. Targeted gray literature focused on how leaders can foster communication within teams, and the positive impacts of an open and accountable culture on staff wellbeing and productivity [73, 74]. These findings echo research on resilient health systems emphasizing that ‘over-managing’ restricts the adaptive capacities needed by teams within dynamic healthcare environments [100, 103]. The literature pointed to the need for leaders to strengthen communication and collaboration at varying levels – environmental, team, and organizational – to enable more efficient and better-quality healthcare delivery, and during this process they should endeavor to model the balance between autonomy and accountability [104]. Implementing regular touchpoints that engage multiple stakeholders, such as communities of practice, can help to create positive feedback loops that enable systems change [105], and overcome organizational barriers to collaboration and information sharing, such as weak relationships and inadequate communication [106, 107]. 

**Self-development and self-awareness** also emerged as an important aspect of leadership. Academic literature focused primarily on how these capacities are developed in leaders through structured education and training, including self-assessments and targeted educational modules [65, 69]. Targeted gray literature discussed a range of activities outside of structured training (e.g., experiential learning) that can support leaders’ self-reflection and development, for physician leaders in particular to assess their performance and improve their leadership approaches [91, 92]. These findings suggest that personal leadership development must go beyond formal curriculum requirements to incorporate everyday learning inputs [78], and align with other recent literature suggesting that self-regulation in leaders can be fostered through practicing self-discipline, boundary-setting, and managing disruptions, particularly in the digital age [108, 109]. Practicing self-awareness can help leaders not only to sense-make in complex systems – to adapt to new situations and make appropriate trade-offs – but also to sense-give – to articulate and express the organization’s vision [40]. A minor theme, observed only in the targeted gray literature, was related to leaders’ roles and competencies in **consumer engagement and advocacy**. The importance of increasing consumer engagement in healthcare was emphasized, as well as the structures that are needed to facilitate these changes [79]. Working alongside consumers was highlighted as critical during times of changing care and need, such as during COVID-19 [77, 86]. Although the involvement of consumers and the public in the co-production of care is increasing [110], there is limited academic literature focused on the roles of leaders in creating optimal environments for co-production. Consumer and community involvement in change efforts helps to improve care processes and outcomes [111], but leaders might face challenges understanding and operationalizing local engagement mechanisms [112]. Identifying the organizational and system levers that enable greater consumer involvement, and how leaders can advocate for these levers in their local context, is a fruitful area for future investigation.

The findings of the current review have implications for professional organizations that train healthcare leaders, such as the Australian College of Health Services Management (ACHSM) in Australia, and train clinicians to be leaders, including the UK’s FMLM. Creating a future-focused curriculum addressing the competencies related to the themes identified, in particular innovation and adaptability, is essential to prepare healthcare leaders for growing and changing scopes of responsibility. Such competencies are less amenable to formal theoretical teaching solely and require carefully crafted experiential learning programs in health settings, with supervision by experienced and effective healthcare leaders.

### Strengths and limitations

A notable strength of this scoping review was the inclusion of a broad range of sources and perspectives on the future of healthcare leadership. We captured empirical studies, theoretical academic contributions (e.g., commentaries from healthcare leaders), and targeted grey literature, which is often a more useful source of information on emerging topics [52]. As a result, our findings identified key future trends in the roles and competencies of leaders, both clinical and non-clinical, across a wide range of contexts and situations. Another strength of this review was its specific focus on contemporary literature that examined *future trends* in leadership, to inform how leaders can prepare for upcoming challenges, rather than focusing on leadership that was effective in the past.

There are limitations to this review. Our search strategies may not have adequately captured other leadership trends applicable across contemporary healthcare settings or those faced by leaders and teams on the front lines of care [113]. Incorporating search terms related to specific settings, as well as complex systems concepts, may have enabled greater inferences to be made about how unique future challenges require new approaches to the development of healthcare leaders. To scope future-focused research and perspectives, database searches were narrowly restricted, and it is likely that key articles were missed. Targeted gray literature searches represent key thought leaders in healthcare and leadership, and while this enabled relevant information to be efficiently collected, undertaking highly focused searches may have introduced bias associated with geographical area (i.e., the UK) and particular stakeholder groups (e.g., policy-makers) [55]. Our choice to limit the current review to studies reporting in OECD countries further limited generalizability to other settings including in low-income and middle-income countries (LMICs) [1]. 

## Conclusions

The roles and competencies of leaders are deeply enmeshed in, and reflective of, a complex and continuously transforming healthcare system. This research highlights the types of roles and competencies important for leaders facing a myriad of challenges, and the range of contexts and situations in which these types of roles and competencies can be applied. The ways in which roles and competencies manifest is highly contextual, dependent on both role responsibilities and the situational demands of healthcare environments.

### Electronic supplementary material

Below is the link to the electronic supplementary material.

Supplementary Material 1

## Data Availability

Data supporting these research findings are available upon reasonable request. Further inquiries can be directed to the corresponding author.

## Abbreviations

FMLM: Faculty of Medical Leadership and Management
NHS: National Health Service
PRISMA-ScR: Preferred Reporting Items for Systematic Reviews and Meta-Analyses Extension for Scoping Reviews
RACMA: Royal Australasian College of Medical Administrators
USA: United States of America
UK: United Kingdom

## References

[1] Figueroa CA, Harrison R, Chauhan A (2019). Priorities and challenges for health leadership and workforce management globally: a rapid review. BMC Health Serv Res.

[2] Braithwaite J, Mannion R, Matsuyama Y (2018). The future of health systems to 2030: a roadmap for global progress and sustainability. Int J Qual Health Care.

[3] Anklam E, Bahl MI, Ball R (2022). Emerging technologies and their impact on regulatory science. Exp Biol Med.

[4] Maeda A, Socha-Dietrich K. Skills for the future health workforce: preparing health professionals for people-centred care. OECD Health Working Papers. Paris: OECD Publishing,; 2021.

[5] Reich MR, Javadi D, Ghaffar A (2016). Introduction to the special issue on effective leadership for health systems. Health Syst Reform.

[6] Shaw J, Brewer LC, Veinot T (2021). Recommendations for health equity and virtual care arising from the COVID-19 pandemic: narrative review. JMIR Form Res.

[7] Graham R, Woodhead T (2021). Leadership for continuous improvement in healthcare during the time of COVID-19. Clin Radiol.

[8] Parr JM, Teo S, Koziol-McLain J (2021). A quest for quality care: exploration of a model of leadership relationships, work engagement, and patient outcomes. J Adv Nurs.

[9] Ree E, Wiig S (2020). Linking transformational leadership, patient safety culture and work engagement in home care services. Nurs Open.

[10] Wu X, Hayter M, Lee AJ (2020). Positive spiritual climate supports transformational leadership as means to reduce nursing burnout and intent to leave. J Nurs Manag.

[11] West M, Armit K, Loewenthal L (2015). Leadership and leadership development in health care: the evidence base.

[12] Churruca K, Ellis LA, Pomare C (2021). Dimensions of safety culture: a systematic review of quantitative, qualitative and mixed methods for assessing safety culture in hospitals. BMJ Open.

[13] Smallwood N, Bismark M, Willis K. Burn-out in the health workforce during the COVID-19 pandemic: opportunities for workplace and leadership approaches to improve well-being. BMJ Lead. 2023;leader–2022. 10.1136/leader-2022-000687 10.1136/leader-2022-000687 PMC12171421 37192091

[14] Kelly RJ, Hearld LR (2020). Burnout and leadership style in behavioral health care: a literature review. J Behav Health Serv Res.

[15] Hutchinson K, Ryder T, Coleman H (2023). Determining the role and responsibilities of the community epilepsy nurse in the management of epilepsy. J Clin Nurs.

[16] Mianda S, Voce A (2018). Developing and evaluating clinical leadership interventions for frontline healthcare providers: a review of the literature. BMC Health Serv Res.

[17] Veronesi G, Kirkpatrick I, Vallascas F (2013). Clinicians on the board: what difference does it make?. Soc Sci Med.

[18] Ferreira TDM, de Mesquita GR, de Melo GC (2022). The influence of nursing leadership styles on the outcomes of patients, professionals and institutions: an integrative review. J Nurs Manag.

[19] Boamah SA (2019). Emergence of informal clinical leadership as a catalyst for improving patient care quality and job satisfaction. J Adv Nurs.

[20] Dickinson H, Ham C (2008). Engaging doctors in leadership: review of the literature.

[21] Hershkovich O, Gilad D, Zimlichman E (2016). Effective medical leadership in times of emergency: a perspective. Disaster Mil Med.

[22] Stoller JK (2009). Developing physician-leaders: a call to action. J Gen Intern Med.

[23] Warren OJ, Carnall R (2011). Medical leadership: why it’s important, what is required, and how we develop it. Postgrad Med J.

[24] Griffith R (1983). NHS management inquiry: report to the secretary of state for social services.

[25] Braithwaite J. Changing how we think about healthcare improvement. BMJ. 2018;361. 10.1136/bmj.k2014 10.1136/bmj.k2014 PMC5956926 29773537

[26] Clay-Williams R, Ludlow K, Testa L (2017). Medical leadership, a systematic narrative review: do hospitals and healthcare organisations perform better when led by doctors?. BMJ Open.

[27] Nieuwboer MS, van der Sande R, van der Marck MA (2019). Clinical leadership and integrated primary care: a systematic literature review. Eur J Gen Pract.

[28] Dwyer AJ (2010). Roles, attributes and career paths of medical administrators in public hospitals: survey of victorian metropolitan directors of Medical services. Aust Health Rev.

[29] Dwyer AJ (2010). Medical managers in contemporary healthcare organisations: a consideration of the literature. Aust Health Rev.

[30] Health Workforce Australia (2013). Health LEADS Australia: the Australian health leadership framework.

[31] NHS Institute for Innovation and Improvement and Academy of Medical Royal Colleges (2010). Medical leadership competency framework: enhancing engagement in medical leadership.

[32] National Centre for Healthcare Leadership. NCHL Health Leadership Competency Model 3.0. National Centre for Healthcare Leadership. 2022. https://www.nchl.org/research/. Accessed 28 December, 2023.

[33] NHS Leadership Academy (2013). Healthcare Leadership Model: the nine dimensions of leadership behaviour.

[34] Frank JR, Snell L, Sherbino J, et al. CanMEDS 2015 Physician Competency Framework. Royal College of Physicians and Surgeons of Canada; 2015.

[35] Dath DCM-K, Anderson G, Burke A, Razack S, Lieff S, Moineau G, Chiu A, Ellison P. Leader. In: Frank JRSL, Sherbino J, editors. CanMEDS 2015 Physician Competency Framework. Royal College of Physicians and Surgeons of Canada; 2015.

[36] Royal Australasian College of Medical Administrators (2011). Medical Leadership and Management Curriculum Framework.

[37] Best S, Stark Z, Brown H (2020). The leadership behaviors needed to implement clinical genomics at scale: a qualitative study. Genet Med.

[38] Thoma B, Karwowska A, Samson L (2023). Emerging concepts in the CanMEDS physician competency framework. Can Med Educ J.

[39] Ahern S, Loh E. Leadership during the COVID-19 pandemic: building and sustaining trust in times of uncertainty. BMJ Lead. 2020;leader–2020. 10.1136/leader-2020-000271

[40] Braithwaite J, Churruca K, Hibbert P, et al. At the heart of getting things done in complex healthcare ecosystems: leadership, strategy, sensemaking and sensegiving. In: Chambers N, editor. Research Handbook on Leadership in Healthcare. Edward Elgar Publishing; 2023. pp. 775–92.

[41] Braithwaite J, Fisher G (2024). Beyond the aspirational: creating the future of health care in Australia. Int Med J.

[42] Cernega A, Nicolescu DN, Meleșcanu Imre M (2024). Volatility, uncertainty, complexity, and ambiguity (VUCA) in healthcare. Healthc.

[43] Kasula BY. Revolutionizing healthcare delivery: innovations and challenges in supply chain management for improved patient care. Trans Latest Trends Health Sect. 2023;15(15).

[44] Niaz M, Nwagwu U (2023). Managing healthcare product demand effectively in the post-covid-19 environment: navigating demand variability and forecasting complexities. Am J Econ Manag Bus.

[45] Thirumalai S, Devaraj S (2024). Mitigating the curse of complexity: the role of focus and the implications for costs of care. J Oper Manag.

[46] Savage M, Savage C, Brommels M (2020). Medical leadership: boon or barrier to organisational performance? A thematic synthesis of the literature. BMJ Open.

[47] Ahti M, Taipale-Walsh L, Kuha S (2023). Health-care leaders’ experiences of the competencies required for crisis management during COVID-19: a systematic review of qualitative studies. Leadersh Health Serv.

[48] Krasikova DV, Green SG, LeBreton JM (2013). Destructive leadership: a theoretical review, integration, and future research agenda. J Manag.

[49] Pellegrini MM, Ciampi F, Marzi G (2020). The relationship between knowledge management and leadership: mapping the field and providing future research avenues. J Knowl Manag.

[50] Paré G, Trudel M-C, Jaana M (2015). Synthesizing information systems knowledge: a typology of literature reviews. Inf Manag.

[51] Heath A, Levay P, Tuvey D (2022). Literature searching methods or guidance and their application to public health topics: a narrative review. Health Info Libr J.

[52] Adams J, Hillier-Brown FC, Moore HJ (2016). Searching and synthesising ‘grey literature’and ‘grey information’in public health: critical reflections on three case studies. Syst Rev.

[53] Tricco AC, Lillie E, Zarin W (2018). PRISMA extension for scoping reviews (PRISMA-ScR): checklist and explanation. Ann Intern Med.

[54] Wolters Kluwer Health I. Get to know the advantages of Ovid MEDLINE^®^. 2024. https://tools.ovid.com/ovidtools/medline.html. Accessed.

[55] Stansfield C, Dickson K, Bangpan M (2016). Exploring issues in the conduct of website searching and other online sources for systematic reviews: how can we be systematic?. Syst Rev.

[56] Ouzzani M, Hammady H, Fedorowicz Z (2016). Rayyan—a web and mobile app for systematic reviews. Syst Rev.

[57] Peters MD, Marnie C, Tricco AC (2020). Updated methodological guidance for the conduct of scoping reviews. JBI Evid Synth.

[58] Arksey H, O’Malley L (2005). Scoping studies: towards a methodological framework. Int J Soc Res Methodol.

[59] Best S, Long J, Stark Z (2019). Clinical leadership and genomics - what works?. Twin Res Hum Genet.

[60] Crabtree BF, Howard J, Miller WL (2020). Leading innovative practice: leadership attributes in LEAP practices. Milbank Q.

[61] Woiceshyn J, Huq JL, Kannappan S (2022). We need to work differently in a crisis: peer-professional leadership to redesign physicians’ work. BMJ Lead.

[62] Appelbaum NP, Liang C, Whitney SE (2022). What business competencies are needed for the modern physician leader and when. Physician Leadersh J.

[63] Hopkins J, Fassiotto M, Ku MC (2018). Designing a physician leadership development program based on effective models of physician education. Health Care Manage Rev.

[64] Sandberg DS, Pennington CM, Lindquist MA (2022). Virtual leadership: CEOs and C-Level executives of healthcare organizations in the United States reimagined new roles as virtual leaders. J Leadersh Stud.

[65] Satiani B, Dawson K, Mehta LS (2022). Lessons learned after implementing an academic faculty leadership program over seven years. Physician Leadersh J.

[66] Angood PB (2022). Transitions and transformations — our choice?. Physician Leadersh J.

[67] Brace R, James D (2020). Adapting to changing roles. J Healthc Manag.

[68] Wright C, Pister K (2018). New thinking leads to better transitions. Physician Leadersh J.

[69] Ramirez B, West DJ, Ramirez CL (2021). Healthcare and leadership competencies, training methods, and venues. J Leadersh Acc Ethics.

[70] Wain MJ, Handel DA, Williford K (2020). Transitioning from physician to hospital leader: a competency-based model. Physician Leadersh J.

[71] Borgwardt HL, Botz CT, Doppler JM (2019). Electronic health record implementation: the people side of change. Manage Healthc.

[72] Anandaciva S. The biggest hospital in England. *The King’s Fund*. 2020. https://www.kingsfund.org.uk/blog/2020/09/biggest-hospital-england. Accessed 25 February, 2023.

[73] Bailey S, West M. Learning from staff experiences of Covid-19: let the light come streaming in. *The King’s Fund*. 2020. https://www.kingsfund.org.uk/blog/2020/06/learning-staff-experiences-covid-19. Accessed 26 February, 2023.

[74] Bailey S, West M. Naming the issue: chronic excessive workload in the NHS. *The King’s Fund*. 2021. https://www.kingsfund.org.uk/blog/2021/06/naming-issue-chronic-excessive-workload-nhs. Accessed 26 February, 2023.

[75] Bhopal A, Hood G. From disaster preparedness to digital disruption: A whistle-stop tour through public health. Faculty of Medical Leadership and Management. 2018. https://www.fmlm.ac.uk/news-opinion/from-disaster-preparedness-to-digital-disruption-a-whistle-stop-tour-through-public. Accessed 27 February, 2023.

[76] Cheetham J. How Leaders in Healthcare 2020 gave an enlightened spotlight to junior doctor leadership. Faculty of Medical Leadership and Management. 2021. https://www.fmlm.ac.uk/news-opinion/how-leaders-in-healthcare-2020-gave-an-enlightened-spotlight-to-junior-doctor. Accessed 27 February, 2023.

[77] Collins B. Leadership and innovation during Covid-19: lessons from the Cardiff and Vale Health System. *The King’s Fund*. 2021. https://www.kingsfund.org.uk/blog/2021/05/leadership-innovation-covid-19-cardiff-vale. Accessed 27 February, 2023.

[78] Cottey L. The trainee and the team. Faculty of Medical Leadership and Management. 2018. https://www.fmlm.ac.uk/news-opinion/the-trainee-and-the-team. Accessed 28 February, 2023.

[79] Dixon M. Engaging the public in designing the future for the NHS. *The King’s Fund*. 2018. https://www.kingsfund.org.uk/blog/2018/01/engaging-public-designing-future-nhs. Accessed 28 February, 2023.

[80] Leveson D. Developing clinical leaders through innovation. *The King’s Fund*. 2021. https://www.kingsfund.org.uk/blog/2021/03/developing-clinical-leaders-through-innovation. Accessed 25 February, 2023.

[81] Naylor C. Getting to grips with climate change: how can the NHS get to net zero? *The King’s Fund*. 2020. https://www.kingsfund.org.uk/blog/2020/01/climate-change-nhs-net-zero. Accessed 19 February, 2023.

[82] Tweedie J. Leadership - so what is it anyway? February, 2018. https://www.fmlm.ac.uk/news-opinion/blog/leadership-so-what-is-it-anyway

[83] Faculty of Medical Leadership and Management. FMLM response to HEE’s Call for Evidence on the Future Doctor. Faculty of Medical Leadership and Management. 2019. https://www.fmlm.ac.uk/news-opinion/fmlm-response-to-hees-call-for-evidence-on-the-future-doctor. Accessed 23 February, 2023.

[84] Faculty of Medical Leadership and Management. Supporting and developing a leading resource for the future. Faculty of Medical Leadership and Management. 2021. https://www.fmlm.ac.uk/news-opinion/supporting-and-developing-a-leading-resource-for-the-future. Accessed 27 February, 2023.

[85] Faculty of Medical Leadership and Management. Support, wellbeing and leadership. Faculty of Medical Leadership and Management. 2021. https://www.fmlm.ac.uk/news-opinion/support-wellbeing-and-leadership. Accessed 27 February, 2023.

[86] Faculty of Medical Leadership and Management. Reflections from the International Healthcare Leadership Conference. 2022. Faculty of Medical Leadership and Management. 2022. https://www.fmlm.ac.uk/news-opinion/reflections-from-the-international-healthcare-leadership-conference-2022. Accessed 27 February, 2023.

[87] Lakhani M, Osuji N, Lees P et al. FMLM statement: racism and good leadership are incompatible. Faculty of Medical Leadership and Management. 2020. https://www.fmlm.ac.uk/news-opinion/fmlm-statement-racism-and-good-leadership-are-incompatible. Accessed 28 February, 2023.

[88] Dougall D, Ross S. Transformational change in health and care: reports from the field. 2018. Accessed February, 2023. The King’s Fund.

[89] Fogden R, Buck D, Franklin B et al. Poverty and the health and care system: the role of data and partnership in bringing change. *The King’s Fund*. 2022. https://www.kingsfund.org.uk/publications/poverty-health-care-system-data-partnership. Accessed 27 February, 2023.

[90] Ham C, Murray R. The NHS 10-year plan: how should the extra funding be spent. *The King’s Fund*. 2018. https://www.kingsfund.org.uk/publications/nhs-10-year-plan. Accessed 28 February, 2023.

[91] Swanwick T, McKimm J. The assessment of leadership development in the medical undergraduate curriculum: a consensus statement. Faculty of Medical Leadership and Management. 2020. https://www.fmlm.ac.uk/resources/the-assessment-of-leadership-development-in-the-medical-undergraduate-curriculum-a. Accessed 22 February, 2023.

[92] Evans P. A radical upward shift to benefit members and fellows. Faculty of Medical Leadership and Management. 2020. https://www.fmlm.ac.uk/news-opinion/a-radical-upward-shift-to-benefit-members-and-fellows. Accessed 22 February, 2023.

[93] Lakhani M. Black History Month and why it matters. Faculty of Medical Leadership and Management. 2021. https://www.fmlm.ac.uk/news-opinion/black-history-month-and-why-it-matters. Accessed 28 February, 2023.

[94] Moralee S, Exworthy M, Rajasingam D et al. What next for medical leaders? Faculty of Medical Leadership and Management. 2018. https://www.fmlm.ac.uk/resources/what-next-for-medical-leaders. Accessed 25 February, 2023.

[95] Warren S, Laverty A. Leading with compassion: supporting the health and wellbeing of NHS staff. *The King’s Fund*. 2022. https://www.kingsfund.org.uk/audio-video/podcast/leading-compassion-health-wellbeing-nhs-staff. Accessed 22 February, 2023.

[96] Hancock M. Matt Hancock at The King’s Fund annual conference 2018. 2018. https://www.kingsfund.org.uk/audio-video/matt-hancock-kings-fund-annual-conference-2018. Accessed.

[97] Olsson-Brown A, Interview with Dr Anna Olsson-Brown - Chair of the Academy of Medical Royal Colleges Trainee Group. Faculty of Medical Leadership and Management. 2020. https://www.fmlm.ac.uk/news-opinion/interview-with-dr-anna-olsson-brown-chair-of-the-academy-of-medical-royal-colleges. Accessed 19 February, 2023.

[98] Denicolai S, Previtali P (2023). Innovation strategy and digital transformation execution in healthcare: the role of the general manager. Technovation.

[99] Borges do Nascimento IJ, Abdulazeem H, Vasanthan LT (2023). Barriers and facilitators to utilizing digital health technologies by healthcare professionals. NPJ Digit Med.

[100] Braithwaite J, Churruca K, Ellis LA (2017). Complexity science in healthcare: aspirations, approaches, applications, and accomplishments. A white paper.

[101] Coiera E. Why system inertia makes health reform so difficult. BMJ. 2011;34210.1136/bmj.d3693 10.1136/bmj.d3693 21700652

[102] Clay-Williams R, Travaglia J, Hibbert P (2017). Clinical Governance Framework. A Report prepared for the Royal Australasian College of Medical Administrators (RACMA).

[103] Plsek PE, Wilson T (2001). Complexity, leadership, and management in healthcare organisations. BMJ.

[104] van Diggele C, Burgess A, Roberts C (2020). Leadership in healthcare education. BMC Med Educ.

[105] Golden RE, Klap R, Carney DV (2021). Promoting learning health system feedback loops: experience with a VA practice-based research network card study. Healthc.

[106] Smith CL, Fisher G, Dharmayani PNA (2024). Progress with the Learning Health System 2.0: a rapid review of Learning Health Systems’ responses to pandemics and climate change. BMC Med.

[107] Hunt RC, Struminger BB, Redd JT (2021). Virtual peer-to-peer learning to enhance and accelerate the health system response to COVID-19: the HHS ASPR Project ECHO COVID-19 Clinical rounds Initiative. Ann Emerg Med.

[108] Mander R, Hellert U, Antoni CH (2021). Self-leadership strategies for coping with flexibility requirements of digital work with a high degree of latitude for time, place and scope for action—a qualitative study. Gr Interakt Org.

[109] Vay C, Steinherr V. Leadership in a digitalized and crisis-ridden world: towards a comprehensive overview of relevant competencies for leaders. University of Hawaiʻi at Mānoa; 2023.

[110] Churruca K, Pomare C, Ellis LA (2021). Patient-reported outcome measures (PROMs): a review of generic and condition‐specific measures and a discussion of trends and issues. Health Expect.

[111] Best A, Greenhalgh T, Lewis S (2012). Large-system transformation in health care: a realist review. Milbank Q.

[112] Bolden R, Kars-Unluoglu S, Jarvis C (2023). Paradoxes of multi-level leadership: insights from an integrated care system. J Change Manag.

[113] Braithwaite J, Dammery G, Spanos S (2023). Learning health systems 2.0: future-proofing healthcare against pandemics and climate change. A White Paper.

