# Supplementary material for: Healthcare leaders navigating complexity: a scoping review of key trends in future roles and competencies
Source: BMC Med Educ. 2024 Jul 3;24:720. doi: 10.1186/s12909-024-05689-4 (PMC11223336; doi:10.1186/s12909-024-05689-4)
Supplement: Supplementary file 1 — Supplementary Material 1 [file 12909_2024_5689_MOESM1_ESM.docx]

**Supplementary file 1**.

Search strategy for OVID MEDLINE

| Healthcare leadership | Physician Executives/td [Trends]  OR ((executive* or leader* or leadership* or manager* or director* or CEO* or board*) adj3 (physician$ or doctor$ or clinician$)).ab.  OR ((collective leader*) or (distributed leader*) or (opinion leader*) or (change agent*)).ab. |
| --- | --- |
|  | AND |
| Competencies and requirements | (competen* OR skill* OR prerequisite* OR requirement* OR prepar* OR knowledg* OR abilit* OR personal* OR educat* OR train* OR communic*).mp. |
|  | AND |
| Healthcare | “Delivery of Health Care”/td [Trends]  OR Health Services/td [Trends]  OR Health Occupations/td [Trends] |
|  | AND |
| Limits | English language  2018 – current |

Search strategy for OVID Embase

| Healthcare leadership | Medical Director/  OR ((executive* or leader* or leadership* or manager* or director* or CEO* or board*) adj3 (physician$ or doctor$ or clinician$)).ab.  OR (((collective leader*) or (distributed leader*) or (opinion leader*) or (change agent*)) adj4 (physician$ or doctor$ or clinician$)).ab. |
| --- | --- |
|  | AND |
| Characteristics / requirements | (competen* OR skill* OR prerequisite* OR requirement* OR prepar* OR knowledg* OR abilit* OR personal* or character* OR attribut* OR educat* OR train* OR communic*).ab. |
|  | AND |
| Healthcare | Health Care Delivery/  Health Services/  Health Occupations/ |
|  | AND |
| Prediction (cannot search by “trends”) | Prediction and Forecasting/  (pattern* OR trend* OR chang* OR shift* OR transform*).ab. |
| Limits | English language  2018 – current |

Search strategy for Business Source Premier

| Healthcare leadership | Healthcare Administration/  OR ((executive* or leader* or leadership* or manager* or director* or CEO* or board*) AND (physician* or doctor* or clinician*)).AB  OR  (((collective leader*) or (distributed leader*) or (opinion leader*) or (change agent*)) AND (physician* or doctor* or clinician*)) |
| --- | --- |
| Characteristics / requirements | (competen* OR skill* OR prerequisite* OR requirement* OR prepar* OR knowledg* OR abilit* OR personal* or character* OR attribut* OR educat* OR train* OR communic*).AB. |
| Healthcare | (healthcare OR "health services").AB |
| Prediction (cannot search by “trends”) | (forecast* OR predict* OR project* OR pattern* OR trend* OR chang* OR shift* OR transform*).AB |
| Limits | English language  2018 – current |
